# Reconstructing Focal Topological Curves through Vortex Spots from Multifocal Metasurfaces

**DOI:** 10.1002/advs.202513605

**Published:** 2025-09-30

**Authors:** Hongguang Dong, Raheel Ahmed Janjua, Zhipeng Hu, Yi Jin, Sailing He

**Affiliations:** ^1^ National Engineering Research Center for Optical Instruments College of Optical Science and Engineering Zhejiang University Hangzhou 310058 P. R. China; ^2^ Department of Electromagnetic Engineering School of Electrical Engineering KTH Royal Institute of Technology Stockholm SE‐100 44 Sweden

**Keywords:** boundary state, focal curve, lattice, multifocal metasurface, vortex spot

## Abstract

Structured light plays an important role in modern photonics. Here, two approaches with distinct properties are proposed for reconstructing focal topological curves using an array of vortex spots generated by synthetic multifocal metasurfaces. A focal vortex lattice can create spatial topological photonics, where the tightly overlapping vortex spots annihilate within the lattice, leaving a closed multi‐contoured boundary state as a closed focal curve defined by the lattice periphery. Alternatively, a chain of tightly overlapping vortex spots directly reconstructs a focal topological curve. The resultant curves are of multi‐contours depending on the vortex topological charge, and the whole may coalesce into an overall vortex if a curve pattern is simple. Multichannel multifocal silicon metasurfaces are designed and fabricated to realize the required focal topological curves, and upconverted fluorescence is used to directly visualize the morphology of the near‐infrared focal fields. This method opens new avenues for high‐dimensional manipulation in focal optical fields with metasurfaces.

## Graphical abstract

Multichannel multifocal silicon metasurfaces are built to reconstruct focal topological curves using an array of vortex spots, and upconverted fluorescence is used to directly visualize the morphology of the near‐infrared focal fields.

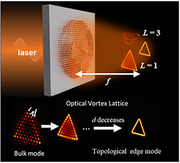

## 1 Introduction

With the rapid advancement of optical manipulation techniques, structured light has emerged as a crucial research focus. Through precise modulation of beam focusing and diffraction properties, researchers can generate intricate spatial light field distributions, including Möbius strips,^[^ ^1^, ^2^, ^3^ ^]^ multi‐knot configurations,^[^ ^4^, ^5^ ^]^ and knotted structures in both scalar and polarization fields.^[^ ^5^, ^6^, ^7^, ^8^, ^9^ ^]^ These breakthroughs hold significant potential for applications in high‐dimensional data storage, enhanced communication bandwidth, information encryption, and robust signal transmission. A particularly promising approach involves discretizing continuous focal curves into spatially distributed focal points, where each focal point functions as an optical “pen” to trace complex spatial patterns. This methodology enables precise spatial organization and controlled movement of particles.^[^ ^10^ ^]^ Furthermore, by strategically arranging these spatial curves with varying structural parameters, researchers have demonstrated remarkable capabilities in pattern holography and advanced information encryption schemes.^[^ ^5^ ^]^ Expanding the controllable dimensions of spatial light fields represents a cutting‐edge research frontier. One particularly viable strategy incorporates angular momentum manipulation during beam propagation, as exemplified by various innovations in vector focal curves^[^ ^11^ ^]^ and longitudinal vortex beams.^[^ ^12^, ^13^, ^14^, ^15^ ^]^

Optical lattices are structured light systems with unique physical properties. Beyond the discretization of planar optical fields into arrays of solid points, optical vortex lattices exhibit physical characteristics arising from the periodic arrangement of vortex atoms carrying quantized orbital angular momenta,^[^ ^16^, ^17^ ^]^ These systems demonstrate exceptional topological properties and dynamic controllability, rendering them invaluable for applications such as optical trapping,^[^ ^18^, ^19^ ^]^ optical communication,^[^ ^5^, ^20^, ^21^, ^22^, ^23^ ^]^ quantum information,^[^ ^24^, ^25^ ^]^ super‐resolution imaging,^[^ ^26^, ^27^ ^]^ and rotation detection.^[^ ^28^, ^29^ ^]^ Topological photonics has emerged as a framework for constructing quantum channels, where topological protection mitigates photon loss and scattering during propagation, while inherent structural robustness enables enhanced tolerance to environmental noise and fabrication imperfections in quantum communication systems. Substantial research efforts have established multiple methodologies for generating vortex lattices with periodic vortex arrays, including but not limited to: far‐field interference of multi‐plane‐wave fronts,^[^ ^30^ ^]^ diffraction of holographic gratings,^[^ ^18^, ^31^ ^]^ pinhole interference,^[^ ^32^ ^]^ the Talbot effect,^[^ ^33^ ^]^ and Damman gratings.^[^ ^34^, ^35^ ^]^ Recent advances, incorporating computational holography with spatial light modulators^[^ ^36^, ^37^, ^38^, ^39^ ^]^ or metasurfaces,^[^ ^40^, ^41^, ^42^, ^43^, ^44^, ^45^ ^]^ have facilitated the creation of vortex lattices comprising heterogeneous vortex atoms with varying topological charges, thereby significantly enhancing information encoding density. While current research predominantly focuses on diffraction‐order modulation to maintain atomic independence within the lattice, the deliberate introduction of coherent interaction presents opportunities for realizing spatial topological photonics (STP), particularly topological boundary states. As fundamental entities in condensed matter physics and topological photonics, boundary states represent robust quantum states or electromagnetic modes localized at interfaces or defects in systems with nontrivial topological invariants.^[^ ^46^ ^]^ They manifest prominently in topological insulators, superconductors, and engineered photonic/phononic crystals,^[^ ^47^ ^]^ emerging when bulk band invariants assume nonzero values. The inherent topological defects in optical vortex phase structures,^[^ ^48^, ^49^, ^50^, ^51^, ^52^ ^]^ provide a viable pathway for realizing optical edge states in free‐space configurations. Some studies enhance interactions between vortex atoms through phase or polarization control, thereby achieving vortex beams with distinctive shapes,^[^ ^53^, ^54^, ^55^, ^56^ ^]^ while crystalline arrangements formed by multiple optical vortex‐anti‐vortex clusters retain their lattice structures over distances spanning several Rayleigh ranges.^[^ ^57^ ^]^ However, there is limited research on the impact of closely adjacent vortex atoms on the overall characteristics.^[^ ^58^ ^]^ This knowledge gap presents significant opportunities for investigating emergent topological phenomena in strongly coupled vortex lattice systems.

In contrast to conventional lens systems, metasurfaces (composed of subwavelength units) enable simultaneous modulation of incident light's amplitude, polarization, phase, and dispersion properties. This capability enhances spatiotemporal control flexibility over light field and expands information capacity. Their Complementary Metal‐Oxide‐Semiconductor (CMOS) compatible fabrication offers compelling advantages for device miniaturization and system integration.

**Figure 1:**
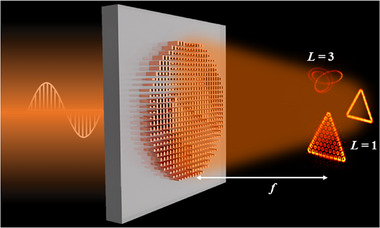
Schematic of a silicon metasurface generating FTCs.

In this study, based on STP and direct discretization, we will reconstruct focal topological curves (FTCs) by an array of focal vortex spots (FVSs) as atoms like that in the case of familiar composite metamaterials. This unlocks the agile wavefront manipulation of metasurfaces (**Figure** **1**). This multifocal platform facilitates dynamic transition between isolated and closely packed vortex configurations. We demonstrate that dense focal vortex lattices (FVLs) exhibit spatial topological photonic phenomena, where interference produces bulk field cancellation and well‐defined boundary states defined by the lattice peripheries, establishing a new reconstruction paradigm. An alternative method directly discretizes focal topological curves (FTCs) into overlapping vortex chains, utilizing topological charges for enhanced morphological control. We thoroughly characterize these systems through numerical modeling and experimental validation based on near‐infrared multiple‐channel multifocal silicon metasurfaces. 3D upconversion fluorescence microscopy complements conventional methods to visualize near‐infrared focal fields.

## 2 Results

### 2.1 Synthetic Multifocal Metasurfaces

**Figure 2:**
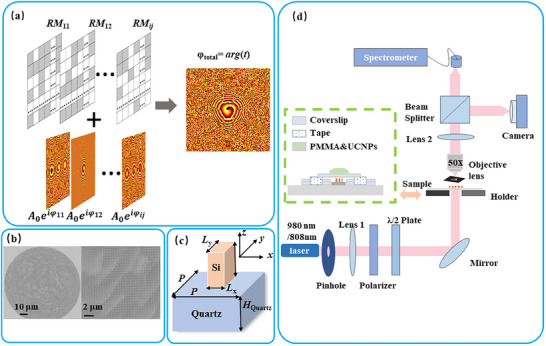
Synthetic multifocal silicon metasurface. a) Construction process of the required phase modulation for the metasurface. Grey areas donate “0” and white areas donate “1” in *RM_ij_^.^* b) Overall image of a fabricated metasurface from a scanning electron microscope (left) with a magnified view of a local area (right). c) Schematic of a metasurface unit. d) Home‐built measurement system.

A synthetic multifocal metasurface exhibits remarkable flexibility in simultaneously generating multiple FVSs (*N* × *M*). It may adopt the following transmission distribution,

(1) $$t\;\left(x,y\right)=\sum_{i=1}^{N}\sum_{j=1}^{M}A_{ij}e^{i\left[\varphi_{\mathrm{lens},ij}\left(x,y\right)+L_{ij}\theta \left(x,y\right)\right]}$$

in which, the subscripts of *i* and *j* designate a particular FVS, *A_ij_* and *L_ij_* represent its amplitude coefficient and topological charge, respectively, *θ*(*x*, *y*) denotes the azimuthal angle on the metasurface, and φ_lens,*ij*_(*x*, *y*) signifies the focusing phase, which is determined by the subsequent formula,

(2) $$\begin{array}{ccc} \varphi_{\mathrm{lens},ij}\left(x,y\right) & = & \frac{2\pi }{\lambda }\left[\sqrt{x_{ij}^{2}+y_{ij}^{2}+z_{ij}^{2}}\right.\\ & & -\left.\sqrt{{\left(x-x_{ij}\right)}^{2}+{\left(y-y_{ij}\right)}^{2}+{\left(z-z_{ij}\right)}^{2}}\right] \end{array}$$

where (*x_ij_*, *y_ij_*, *z_ij_*) denotes the position coordinate of the FVS, (*x*, *y*, *z*) denotes the coordinate position of each metasurface unit (the metasurface plane is fixed at *z* = 0 here), and λ is the working wavelength. Consequently, *z_ij_* in Equation (2) represents the relative longitudinal position of the focal plane on which FVS(*i*, *j*) is located, namely focal length *f* = *z_ij_*. To streamline the design of the metasurface, one can simply extract the phase distribution from Equation (1) by disregarding the amplitude. Therefore, the simplified metasurface must exhibit the following phase distribution to effectively modulate the incident light in numerical calculation,

(3) $$\varphi_{\mathrm{total}}\;\left(x,y\right)=arg\left[\sum_{i}^{N}\sum_{j}^{M}e^{i[\varphi_{\mathrm{lens},ij}\left(x,y\right)+L_{ij}\theta \left(x,y\right)]}RM_{ij}\left(x,y\right)\right]$$

where operator *arg* represents the radial angle of a complex number, *RM_ij_* is a binary mask matrix in which each element labelled by (*x*, *y*) is randomly set between 0 and 1. When generating a vortex lattice, the phase distribution of the metasurface is imposed by some periodicity, which can induce undesirable diffraction, which degrades the focal field. Thus, the introduction of randomness may be required to suppress the higher‐order diffraction while preserving the independence of each focal vortex spot.^[^ ^59^, ^60^ ^]^ This calculation process is depicted in **Figure** **2**a. When a plane wave serves as the illuminating source, the output field of the designed metasurface assumes the following configuration,

(4) $$U_{0}\left(x,y\right)=\mathrm{circ}\left(\frac{r}{R}\right)e^{i\varphi_{\mathrm{total}}\left(x,y\right)}$$

where circ(*r*/*R*) is a truncation function from that a practical metasurface is a finite‐size circular aperture (*R* is the radius of the metasurface and *r* =$\;\sqrt{x^{2}+y^{2}}$). The relationship between the output light field of *U*_0_(*x*,*y*) and the propagated light field of *U*(*x*′, *y*′) reaching an observation plane can be expressed by the following scalar diffraction,

(5) $$u_{0}\left(f_{x},f_{y}\right)=\int \;\;\;\int U_{0}\left(x,y\right)e^{-i2\pi \left(f_{x}x+f_{y}y\right)}\;dxdy$$

(6) $$u\left(f_{x},f_{y}\right)=u_{0}\;\left(f_{x},f_{y}\right)e^{ikz\sqrt{1-{\left(\lambda f_{x}\right)}^{2}-{\left(\lambda f_{y}\right)}^{2}}}$$

(7)

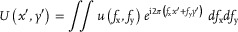

where *k*  =  2π/λ, *u*_0_(*f*_x_,*f*_y_) is the angular spectrum of *U*_0_(*x*,*y*) at the output surface of the metasurface, *u*(*f*_x_,*f*_y_) is that on the observation plane (the separation between the latter and the former is the propagation distance of *z*), (*f*_x_ = cos α/ λ, *f*_y_ = cos β/λ ) is the spatial angular spectrum with cos α and cos β as the directional cosine components of the transverse wave vector. From the above angular spectrum theory of scalar diffraction, we can numerically calculate the diffracted light field of the incident plane wave in the far field after passing through the metasurface.

Based on the designated phase modulation, a corresponding silicon metasurface can be designed and fabricated (see Notes S1 and S2, Supporting Information). The diameter of the fabricated metasurface is 300 µm as shown in Figure 2b and its working wavelength is set at 980 nm. A unit of the metasurface is illustrated in Figure 2c (Note S3, Supporting Information), in which the unit period is *P* = 350 nm, and a silicon brick with height *H* = 420 nm is placed on a 500 µm thick quartz substrate. By adjusting the width of *L*_x_ and length of *L*_y_ of the silicon block, the produced phase response can be varied within the whole range of 0–2π under x‐polarized incidence while high transmittance is sustained. Such phase response can also be independently achieved for y‐polarized incidence. By optimization, one can also nearly independently manipulate the phase response at some other wavelength. Thus, one can achieve a multi‐channel metasurface for multiple focal vortex spots.

### 2.2 FTCs Reconstructed from STP

Each FVS can be established almost independently on its corresponding specified focal plane within Equation (3), thereby providing a flexible capability for arranging an FVL. The lattice can be formed not only on a focal plane but also on a noncoplanar surface. Lattice patterns can be varied widely, including rectangular, trigonal, hexagonal periodic structures, or even quasicrystals. Additionally, each FVS can be assigned a specific topological charge of *L*.

**Figure 3:**
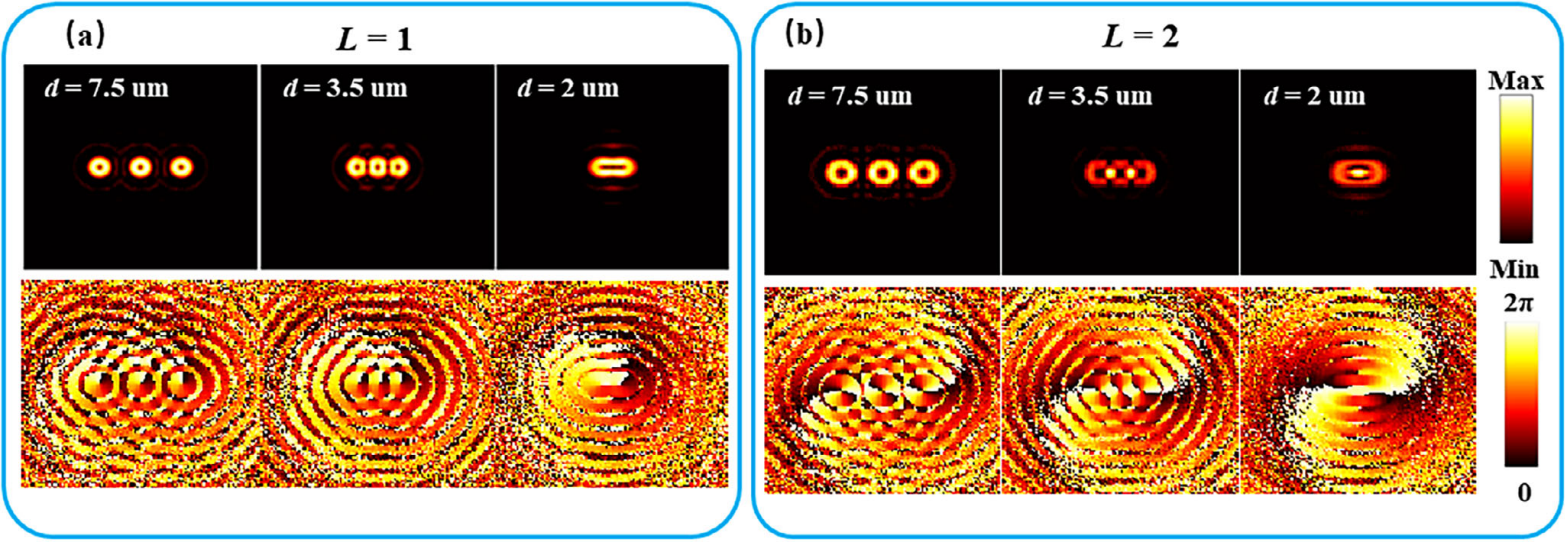
Evolution of the field distribution around a line chain of FVSs as the separation between neighboring spots is reduced gradually. (a) Topological charge of each spot is *L* = 1 and (b) *L* = 2. The top panels are for the electric amplitude distributions on the focal plane (*f* = 400 µm) and the bottom panels for the phase distributions.

Here, we primarily consider the extreme situation, where the FVSs are in a close‐packing arrangement on a focal plane. The theoretical calculation of the focal field refers is discussed in the Experimental Section. At first, a line chain of FVSs with topological charge *L* = 1 is investigated (the focal plane is fixed at *f* = 400 µm far from the metasurface). When the separation of *d* between two neighboring spots is greater than or equal to the size of a single spot (≈5 µm, Note S4, Supporting Information), there is weak interference superposition between adjacent spots so that the hollow spots on the focal plane are discrete as shown in the top left panel of **Figure** **3**a and each one is a vortex individually as indicated by the phase distribution in the bottom left panel of Figure 3a. As *d* is reduced to be less than the diameter of a FVS but greater than the radius, the spots begin to touch each other and lose the role as an individual vortex center gradually, which is displayed by the middle panels in Figure 3a. As *d* is further reduced, the spots are eventually indistinguishable, and the intermediate field along the chain is suppressed by the destructive interference of two neighboring spots which form a phase difference of π at the center between them. Thus, a bright contour is formed surrounding the spot chain as shown by the top right panel in Figure 3a. In the bottom right panel of Figure 3a, one can also see that the spot collection acts as a vortex center of topological charge *L* = 1.

After this, the case of *L* = 2 is also investigated. The varying tendency of the field distribution is similar to that for *L* = 1 as shown by Figure 3b. However, the intermediate field along the chain is enhanced by the constructive interference of each two neighboring spots which form a phase difference of 2π at the center between the them, and a relatively weaker light contour is formed around the spot chain as shown by the top right panel of Figure 3b. Through further investigation, one can find that the field distribution for larger odd *L* behaves similar to the case of *L* = 1, and that for larger even *L* similar to the case of *L* = 2. However, the radius size of a FVS increases following the possessed topological charge, so it is necessary to enlarge the separation distance between neighboring spots for a larger topological charge.

**Figure 4:**
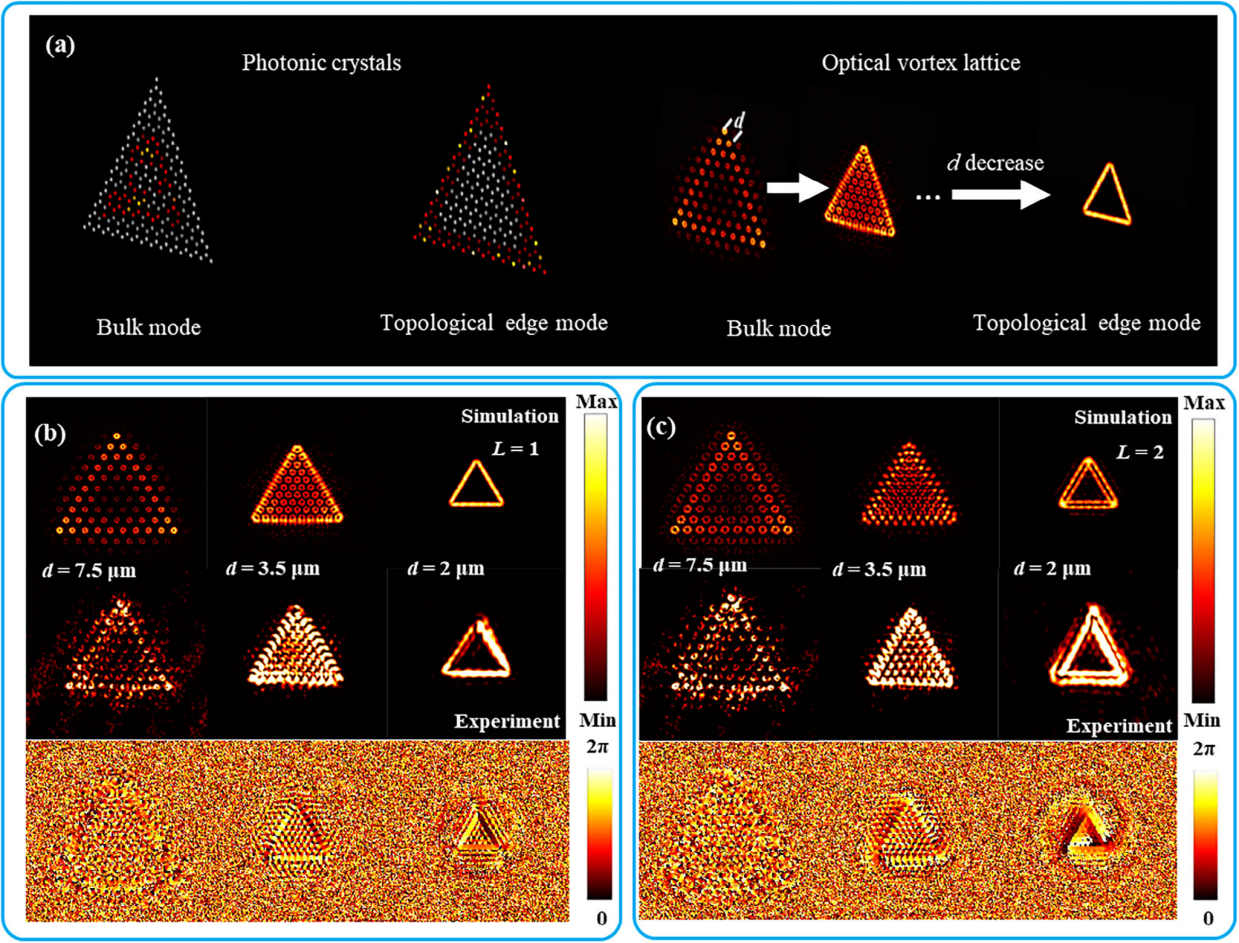
Synthetic FVLs. a) Schematic of the formation of STP. b) Triangular FVL with topological charge *L* = 1. c) Triangular FVL with *L* = 2. b,c) The top and bottom rows are for the simulated electric amplitude and phase distributions, respectively, and the middle row is for the measured field distributions.

Now, FVLs are considered. When the spot separation of *d* is large, each spot in a lattice remains independent, like that in the case of the above line chains. However, when *d* is enough small, the interference between the spots may induce a spatial version of the famous topological photonics as illustrated in **Figure** **4**a. For demonstration, a triangular FVL of *L* = 1 is first constructed on the focal plane of *f* = 400 µm, which is shown in Figure 4b with each long side containing 10 periods. When *d* = 7.5 µm larger than the spot diameter, although one can clearly distinguish the shape of each hollow vortex spot in Figure 4b, interference still appear at some degree inside the lattice so that the central spots are weakened. When *d* = 3.5 µm, the FVSs begin to touch each other. The strength of each spot inside the lattice becomes similar, and a bright continuous contour is formed along the long sides of the lattice. At last, when *d* is further reduced to 2.0 µm, pronounced destructive interference appears inside the lattice. Thus, there is rather weak field inside the lattice and only a bright contour as a boundary state is left around the lattice periphery. According to the bottom row of Figure 4b, each spot is a vortex center at beginning and the spot collection acts as the vortex center with *L* = 1 at last. It should be noted that *d* cannot be too small, or else the STP may be degraded. To confirm the above theoretical prediction, a corresponding metasurface is fabricated, and the measurement system illustrated in Figure 2d is used to record the focal light field. A solid‐state 980 nm laser is connected to the optical path as a light source by a single‐mode fiber. The laser passes through a pinhole and is collimated by a lens behind it through a polarizer to form polarized light. A half‐wave plate is used to change the polarization direction of the incident light. After the specific polarized light passes through the metasurface sample, the light field is collected by a 50x objective and imaged on the camera through the lens. The measured result is shown in the middle row of Figure 4b. The experimental field distributions on the focal plane for different spot separations of *d* are highly consistent with the corresponding simulated results.

Other topological charges are also interesting in forming STP. When the held topological charge of *L* in Figure 4b is varied from 1 to 2, the variation tendency of the field distribution on the focal plane for different spot separations is shown in Figure 4c. For large *d* = 7.5 µm, there is no noticeable difference in the field distribution compared with the case of *L* = 1. However, when *d* = 3.5 µm, the field distribution is more complex, and a bright contour is formed near the outmost side, not at the outmost side. This is induced the vortex property of the spots. When *d* = 2.0 µm, spatial topologic photonics also appear, but the boundary state consists of two bright contours instead of one. In the bottom row of Figure 4c, one can clearly observe a phase vortex of *L* = 2 with the spot collection as the center. The above behavior is also experimentally tested as shown in the middle row of Figure 4c. As the topological charge continues to be enlarged (not very large), STP may still be sustained after adjusting the period separation appropriately, and the spot collection behaves like a vortex. For odd *L*, the boundary state mainly consists of one bright contour like the case of *L* = 1, and for even *L*, it mainly consists of two bright contours like the case of *L* = 2. In all, a line chain of FVSs can be used to understand the behavior of a FVL, but some difference exists between their behaviors.

In this study, we primarily investigate a fundamental phenomenon of STP. While a more complex vortex lattice can indeed support a topological boundary state (Note S5, Supporting Information), the phase vortex center of the spot collection may not persist under such a condition. Our observation reveals that the topological boundary states formed from FVLs enable a unique approach to reconstruct closed FTCs. We will also explore an alternative methodology for reconstructing FTCs.

### 2.3 FTCs Reconstructed from Direct Discretization

A free focal topological curve (FTC) can be discretized into *N* focal vortex spots (FVSs). If these spots are enough closely packed, an approximately continuous curve may be reconstructed. The topological charge possessed by the constituting FVSs determines the cross section of a reconstructed FTC. The line chains of FVS investigated in Figure 3 is the simplest case. Here, FTCs are investigated in depth. Coplanar and 3D noncoplanar curves will be reconstructed.

To better demonstrate the flexibility in engineering FTCs, a multi‐channel multifocal metasurface is designed (Note S3, Supporting Information). It works at two wavelengths of 980 and 808 nm simultaneously, and it performs response to x‐ and y‐ polarized incidences at each working wavelength nearly independently. Thus, there are four channels of which each one can form a special focal curve (Note S6, Supporting Information).

**Figure 5:**
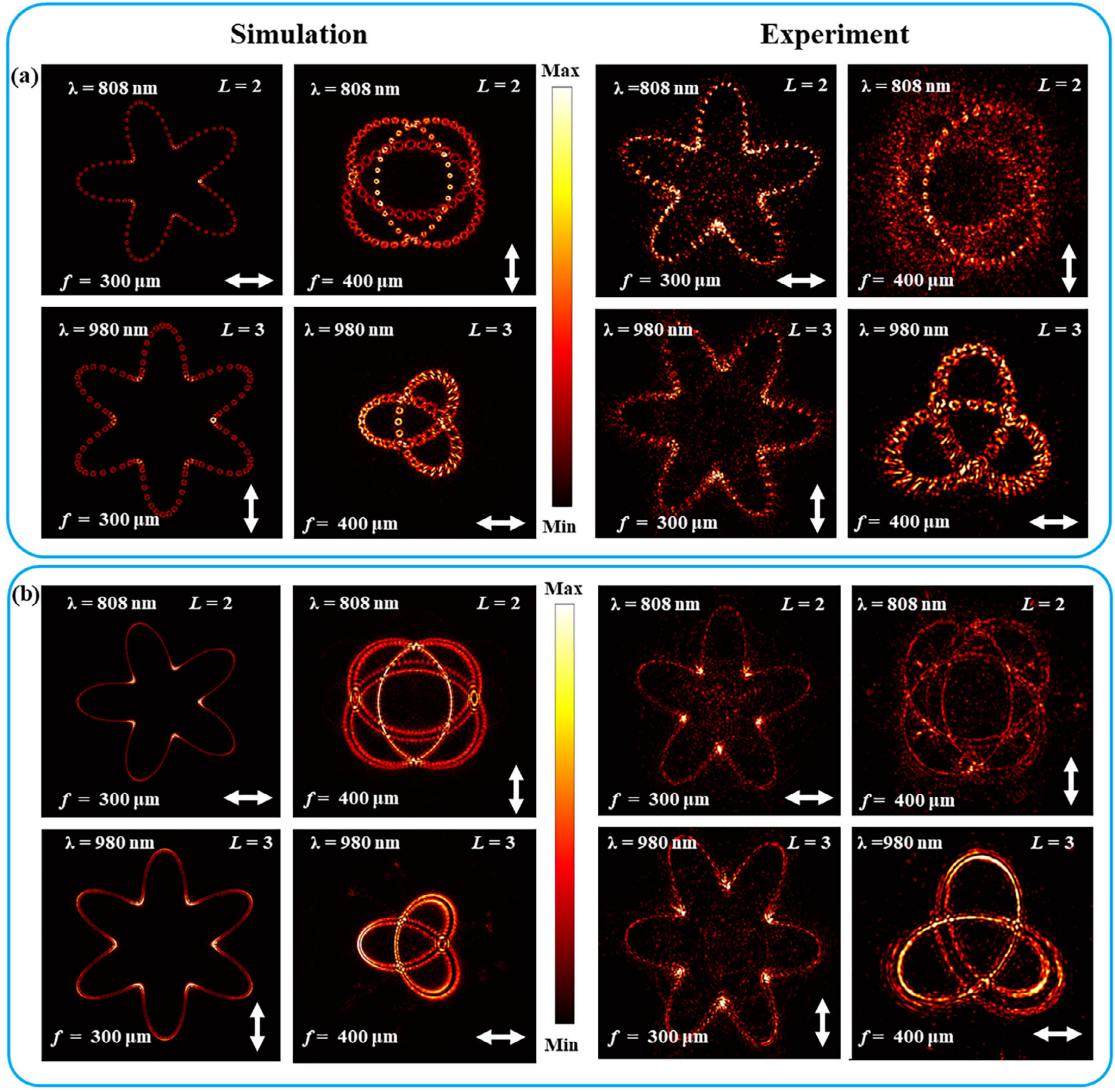
FTCs reconstructed in four metasurface channels. a,b) FTCs linked by *N* = 100 and *N* = 600 vortex spots, respectively. The four channels are indicated in the images. The left and right panels of (a) and (b) are for simulated and experimental results, respectively. The horizontal double‐headed arrows represent x‐polarization, and the vertical ones represent y‐polarization.

At first, the four FTCs are composed of sparse FVSs (*N* = 100). In the left panel of **Figure** **5**a, one can see that at wavelength 808 nm, the x‐polarized channel forms a coplanar pentagram curve linked by discrete hollow vortex spots of *L* = 2 on the focal plane of *f*_1_ = 300 µm, and the y‐polarized channel forms a 3D noncoplanar knotted curve linked by discrete hollow vortex spots of *L* = 2 (each spot has its individual focus length floated in a range of *f*_2_ = 400 ± 15 µm, and the corresponding electric amplitude distribution is recorded on the focal plane of *f*_2_ = 400 µm). Similarly, at wavelength 980 nm, the y‐polarized channel forms a coplanar hexagram curve linked by discrete hollow vortex spots of *L* = 3 at the focal plane of *f*_1_ = 300 µm, and the x‐polarized channel forms a noncoplanar knotted curve linked by discrete hollow vortex spots of *L* = 3 (the focus length of each spot is also floated in a range of *f*_2_ = 400 µm ± 10 µm, and the corresponding electric amplitude distribution is recorded on the focal plane of *f*_2_ = 400 µm). Such a metasurface sustaining such four FTCs is designed and fabricated. The recorded field distributions are shown in the right panel of Figure 5a. One can see that the overall profiles of the four experimental FTCs are in agreement with the simulated ones, respectively, but they are blurred by the noise from the fabrication error of the focusing metasurface at some degree and the influence of the error introduced during the design process (see Notes S7 and S8, Supporting Information). Fabrication errors exert more significant influence on the phase response of the metasurface at wavelength 980 nm than at 808 nm, consequently leading to increased noise. In addition, the beam uniformity of the 808 nm laser, coupled into the system through a multimode fiber, is inferior to that of the 980 nm laser delivered via a single‐mode fiber, which is another factor contributing to the higher speckle noise observed in the images generated by the 808 nm channel.

**Figure 6:**
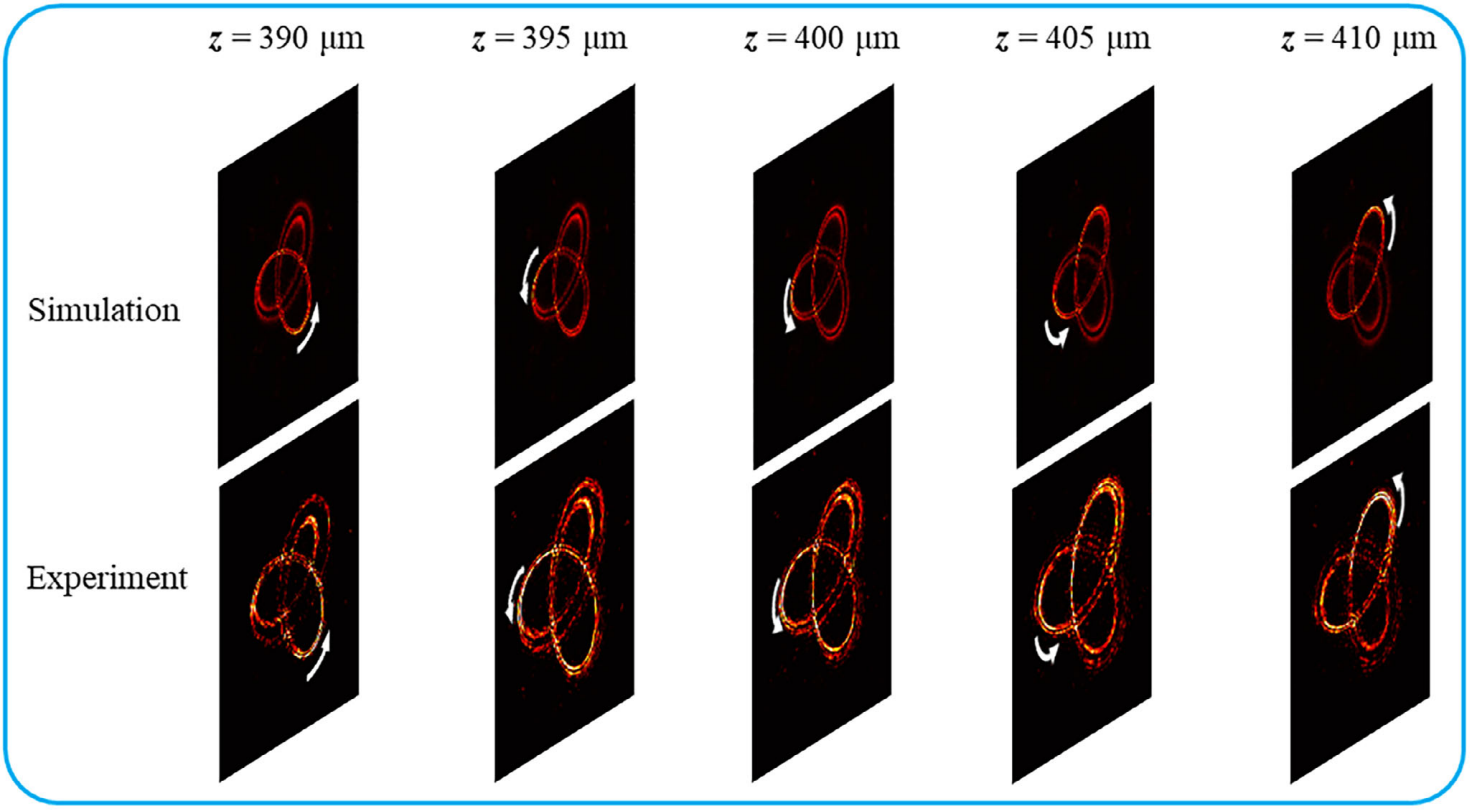
Propagation evolution of a focal knotted curve corresponding to the x channel at wavelength 980 nm in Figure 5b. Five images are snapshotted along the propagation direction. The top images are numerically simulated electric amplitude distributions at different focal planes and the bottom ones are experimentally measured field distributions.

Then, the four FTCs composed of closely packed FVSs (*N* = 600) are investigated, and the simulated results are shown in the left panel of Figure 5b. The spots are enough closely packed so that the separation between neighboring spots is smaller than the spot size, and the spots overlap to form a continuous curve. Depending on the topological charge possessed by the constituting FVSs, each FTC is split into multiple parallel contours. For *L* = 2, an FTC splits into three parallel contours with a bright main contour in the middle and for *L* = 3, an FTC splits into two parallel main contours. The superposition of FVSs along a topological curve resembles the spatial extension of the line chains of FVSa investigated in Figure 2. For moderate odd *L*, it is similar to that of the line chain of FVSs with *L* = 1, and for moderate even *L*, it is similar to that of the line chain of FVSs with *L* = 2. To confirm the simulated prediction, a corresponding metasurface is fabricated and measured. The measured electric field distributions are shown in the right panel of Figure 5b. The overall profiles of the four experimental continuous FTCs are also in agreement with the simulated ones, respectively, although they are also blurred at some degree. In addition, to better disclose the focusing behavior of the 3D focal knotted curve of *L* = 3 at wavelength 980 nm, a series of simulated electric amplitude distributions on different focal planes are recorded and shown in the top of **Figure** **6**. The focal position can be distinctly observed moving along the FTC. The numerical result is consistent with the experimental observation.

### 2.4 Upconversion Fluorescence Imaging of FTCs

**Figure 7:**
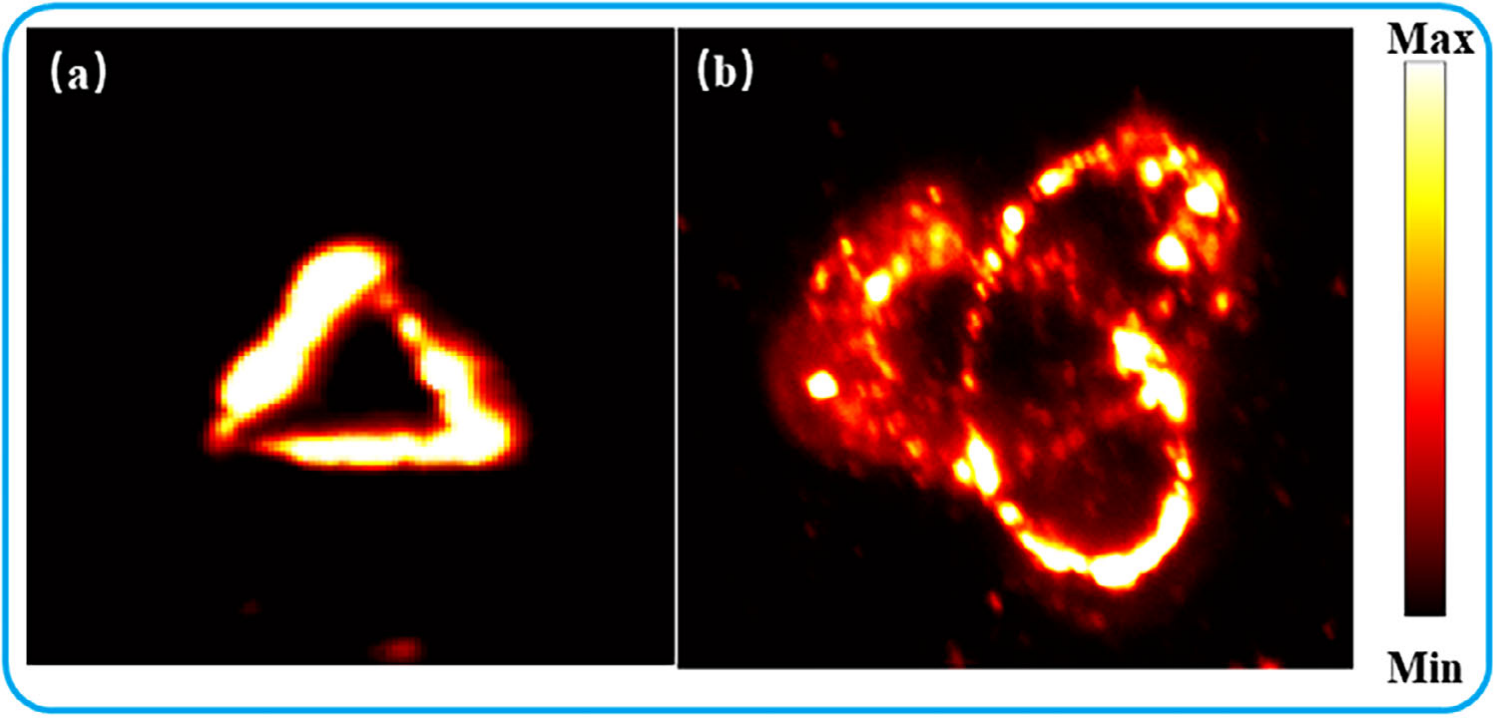
Upconversion fluorescence images excited by FTCs. a) The topological boundary state investigated in Figure 4b is used to excite the upconversion fluorescence. b) The focal knotted curve investigated in Figure 6 is used as the excitation source.

As demonstrated in Note S9 (Supporting Information), upconversion nanoparticles (UCNPs) exhibit dual emission peaks at 540 nm (green) and 660 nm (red) when excited by a polarized 980 nm continuous‐wave laser, enabling direct 3D visualization of near‐infrared focal curves. In our imaging system, a polymethyl methacrylate (PMMA) matrix containing dispersed UCNPs is suspended above the metasurface sample. The 980 nm excitation laser beam, modulated by the metasurface, generates FTCs within the PMMA medium by causing nanoparticles along the curves to emit visible light that precisely maps the focal topological patterns. For experimental validation, we first excite the nanoparticles using the topological boundary state investigated in Figure 3b, and the resulting fluorescence is captured by a color CCD camera simultaneously detecting both emission wavelengths (**Figure** **7**a). The acquired image faithfully reproduces the focal curve's topological pattern, though minor quality degradation occurs due to nanoparticle aggregation and non‐uniform dispersion within the PMMA photoresist. Subsequent excitation with the focal knotted curve in Figure 6 yields similar pattern‐matching fluorescence (Figure 7b). The collection efficiency of fluorescence is inherently associated with the focal position of the objective lens in the optical system while the luminous intensity of fluorescence exhibits a linear proportionality to the optical intensity of the excitation light. The excitation light is focused along a curvilinear trajectory, enabling sequential excitation of UCNPs along the propagation path. When the objective lens is focused on a specific plane along the curvilinear curve, the spatial distribution of fluorescence radiation can be systematically observed and the fluorescence intensity near the observation plane may be most obvious.

## 3 Discussion and Conclusion

We have demonstrated the synthesis of focal vortex lattices (FVLs) and focal topological curves (FTCs) by using focal vortex spots (FVSs) generated by multifocal metasurfaces, establishing a versatile platform for flexible focal‐field engineering. Although the current investigation is limited to scalar cases (x‐ or y‐ polarization), incorporating vectorial FVSs spots could enable vectorial FVLs and FTCs, yielding richer phenomena such as cylindrical vectorial beam arrays with polarization singularity. Regarding STP, the demonstrated boundary states just represent one foundational aspect. Analogous to conventional topological photonics, this framework should accommodate novel phenomena including lattice‐dependent boundary configurations, inter‐lattice boundary states between distinct vortex systems, and intra‐lattice topological defects. The reconstructed FTCs are under planar/near‐planar confinement (narrow depth). Extending the depth requires accounting for the propagation phase into compensation, while axially arranged vortex spot arrays may enable unique curve synthesis through coherent overlapping. The phase‐only metasurfaces used here neglect amplitude modulation, potentially degrading imaging quality and induce parasitic diffraction orders under periodic phase arrangement. Implementing complex‐amplitude response would maximize the synthesis fidelity and enable intensity‐modulated focal curves. While uniform topological charges are employed throughout this work, superimposing varied topological charges could generate more complex optical patterns.

In summary, we have reconstructed FTCs based on vortex spot‐enabled synthesis of programmable focal optical fields via discrete spatial sampling. A FTV can be reconstructed as a boundary state around an arbitrary FVL via the effect of STP besides directly discretizing an FTC. The cross sections of the reconstructed FTCs may be shaped into multiple main contours, and if the curve shape is simple, the spot collection transits into a vortex center. Both depend on the topological charge of the constituting vortex spots. The vortex property of the building FVSs endows us with rich freedom in reconstructing FTCs, of which just a part has been demonstrated as discussed above. The experimental realization of the above investigation is based on synthetic multifocal metasurfaces, which may allow us to achieve several different focal fields at the same time. The performed upconversion imaging directly observes the realized near‐infrared FTCs. Our proposed methods are effective and excellent in reconstructing FTCs. Our methods may inspire applications in various areas, such as particle manipulation, holography, microscopy, sensing, and encryption.

## 4 Experimental Section

### Simulation

The response simulation of a unit structure is based on the rigorous coupled‐wave analysis (RETICOLO V9), where the refractive index of quartz is 1.45, and the refractive index of amorphous silicon is given in Note S1 (Supporting Information). The focal lattices and curves are simulated based on the scalar angular spectrum diffraction in MATLAB.

### Fabrication

The fabrication process follows the flowchart in Note S2 (Supporting Information). Initially, a 420 nm thick amorphous silicon film was grown on a cleaned 500 µm thick 4 in. quartz substrate using plasma‐enhanced chemical vapor deposition (PECVD), and then cut the wafer into 1 cm × 1 cm pieces. The samples were ultrasonically cleaned in acetone for 7 min and in isopropanol for 8 min, rinsed with deionized water, blown dry with nitrogen, and baked on a 100 °C hot plate for 2 min. A 270 nm layer of PMMA (679.04) electron beam resist was spin‐coated onto the prepared samples, baked on a 180 °C hot plate for 10 min, then exposed with an electron beam lithography machine (EBL, Rath150) to create patterns. The samples were then developed in a developer solution (IPA: MIBK = 3:1) for 35 s, and in a fixer solution (IPA) for 35 s, rinsed in water for 35 s, and blown dry with nitrogen. A 20 nm thick chromium (Cr) film was deposited on the sample surface using a magnetron sputtering machine (Kurt J. Lesker PVD 75), and then the sample was ultrasonicated in acetone for 3 min and rinsed with deionized water to obtain patterns with Cr as the mask. The samples obtained after the previous lift‐off process were etched into corresponding nano‐sized rectangular blocks using a dry etching process with C_4_F_8_ and SF_6_ gases (Multiplex ICP STS), and finally, the residual Cr on the top layer was removed using chromium etchant.

### Synthesis of UCNPs

Yttrium acetate hydrate (Y(CH_3_COO)_3_·*x*H_2_O, 99.9%), ytterbium acetate hydrate (Yb(CH_3_COO)_3_·*x*H_2_O, 99.9%), erbium acetate hydrate (Er(CH_3_COO)_3_·*x*H_2_O, 99.9%), ammonium fluoride (NH_4_F, 98%), sodium hydroxide (NaOH, ≥96%), oleic acid (90%), and 1‐octadecene (90%) were from Sigma‐Aldrich. Cyclohexane, methanol, and absolute ethanol were from Sinopharm Chemical Reagent Company. All the reagents were of analytical grade and used without further purification.

For the synthesis of NaYF_4_:Yb, 20%, Er, 21% upconversion nanocrystals, 0.590 mmol of yttrium acetate, 0.200 mmol of ytterbium acetate, and 0.210 mmol of erbium acetate were dissolved in 8 mL of oleic acid in a 50 mL three‐necked flask and heated to 150 °C for 30 min to remove residual water. After dehydration, 12 mL of 1‐octadecene was added, and the mixture was maintained at 150 °C for another 30 min until a clear solution formed, then cooled to room temperature. 10 mL methanol solution containing 4 mmol of ammonium fluoride and 2.5 mmol of sodium hydroxide was then added, and the mixture was stirred at 60 °C for 30 min. After the methanol was evaporated, the reaction mixture is heated under nitrogen to 300 °C and maintained for 90 min to promote nanocrystal growth, followed by natural cooling to room temperature. The product was precipitated by the addition of ethanol, collected by centrifugation, washed three times with ethanol, and finally dispersed in 4 mL of cyclohexane for further use. This procedure yields well‐defined NaYF_4_:Yb, 20%, Er, 21% upconversion nanocrystals.

### Mixture of UCNPs and PMMA

0.1 mg of synthesized UCNPs are mixed with 1.5 mL of cyclopentanone and 1.5 mL of PMMA, followed by ultrasonication for 10 minutes using an ultrasonic cleaner. The mixture is then left to stand to allow the cyclopentanone to evaporate.

### Optical Characterization

The optical measurement system is shown in Figure 2d. The 980 nm continuous laser generated by a solid‐state laser is coupled into free space through a single‐mode fiber. After passing through a pinhole aperture, the beam is collimated by a lens and directed into the system. A polarizer and a half‐wave plate are used to control the polarization direction of the incident light. A 50x objective lens is used to collect the imaging patterns and fluorescence holograms modulated by the metasurface, and the imaging information is recorded by a camera. When measuring the fluorescence holograms, four 120 µm thick coverslips are fixed onto a slide with tape to form supports at a certain distance apart. The metasurface sample is placed between the two supports, and another coverslip of the same thickness is placed over the supports to protect the metasurface sample, which is then fixed with tape. During measurement, the PMMA solution mixed with UCNPs is dripped onto the top cover glass, and a spectrometer is used to collect the fluorescence information from the upconversion nanoparticles. A short‐pass filter with a cutoff wavelength of 750 nm is used to filter out the excitation light.

## Conflict of Interest

The authors declare no conflict of interest.

## Supporting information



Supporting Information

## Data Availability

Data underlying the results are available from the corresponding authors upon request.
