# Supplementary material for: Reconstructing Focal Topological Curves through Vortex Spots from Multifocal Metasurfaces
Source: Adv Sci (Weinh). 2025 Sep 30;12(48):e13605. doi: 10.1002/advs.202513605 (PMC12752623; doi:10.1002/advs.202513605)
Supplement: Supplementary file 1 — Supporting Information [file ADVS-12-e13605-s001.docx]

**Supporting Information**

**Reconstructing Focal Topological Curves through Vortex Spots from Multifocal Metasurfaces**

*Hongguang Dong, Raheel Ahmed Janjua, Zhipeng Hu, Yi Jin* and Sailing He**

**Supplementary Note 1: Refractive index of amorphous silicon**

**Supplementary Figure 1**. Refractive index of amorphous silicon measured by ellipsometry.

**Supplementary Note 2: Fabrication process of a silicon metasurface**

**
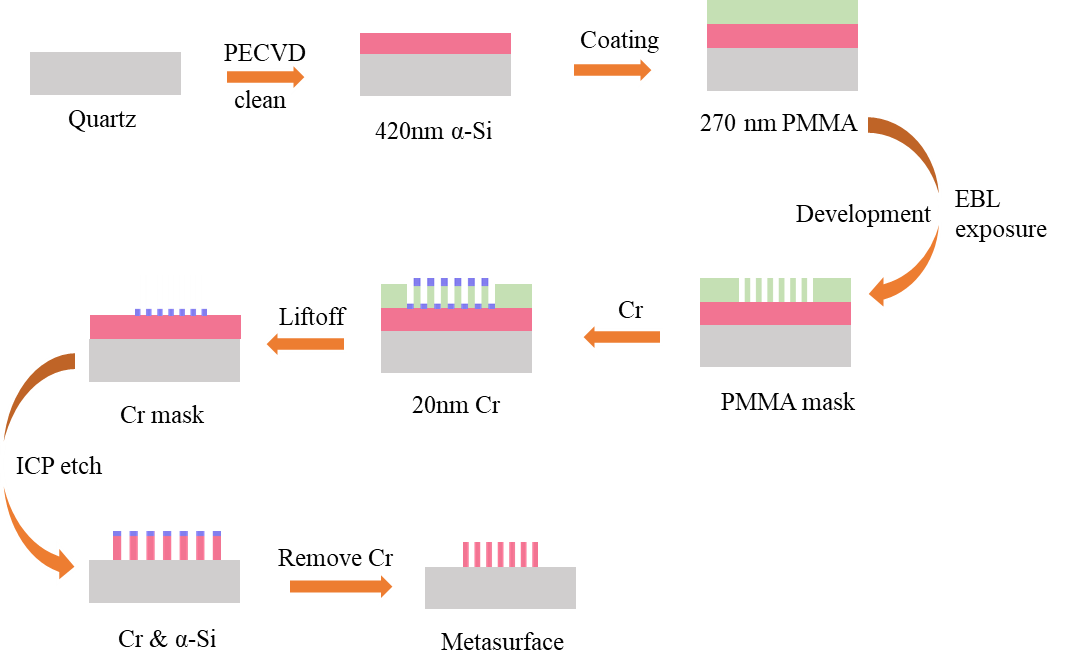
**

**Supplementary Figure 2.** Fabricating a silicon metasurface. First, a 420 nm thick amorphous silicon layer is deposited on a quartz substrate via PECVD. Next, a 270 nm layer of PMMA (product no. 679.04) electron beam resist is spin-coated onto a sample, baked on a 180 °C hot plate for 10 minutes. Subsequently, a pattern is exposed using an EBL system (Rath150). The sample is then developed in a developer solution (IPA: MIBK = 3:1) for 35 seconds, followed by immersion in a fixer solution (IPA) for 35 seconds, and rinsed in water for 35 seconds. Afterward, a 20 nm thick Cr film is deposited via magnetron sputtering (Kurt J. Lesker PVD 75). The sample is ultrasonicated in acetone for 3 minutes and rinsed with deionized water to obtain Cr-masked patterns. Finally, a dry etching process using C_4_F_8_ and SF_6_ gases (Multiplex ICP STS) etches the sample into nano-sized rectangular blocks, and the residual Cr is removed with chromium etchant.

**Supplementary Note 3: Transmission response of metasurface units**


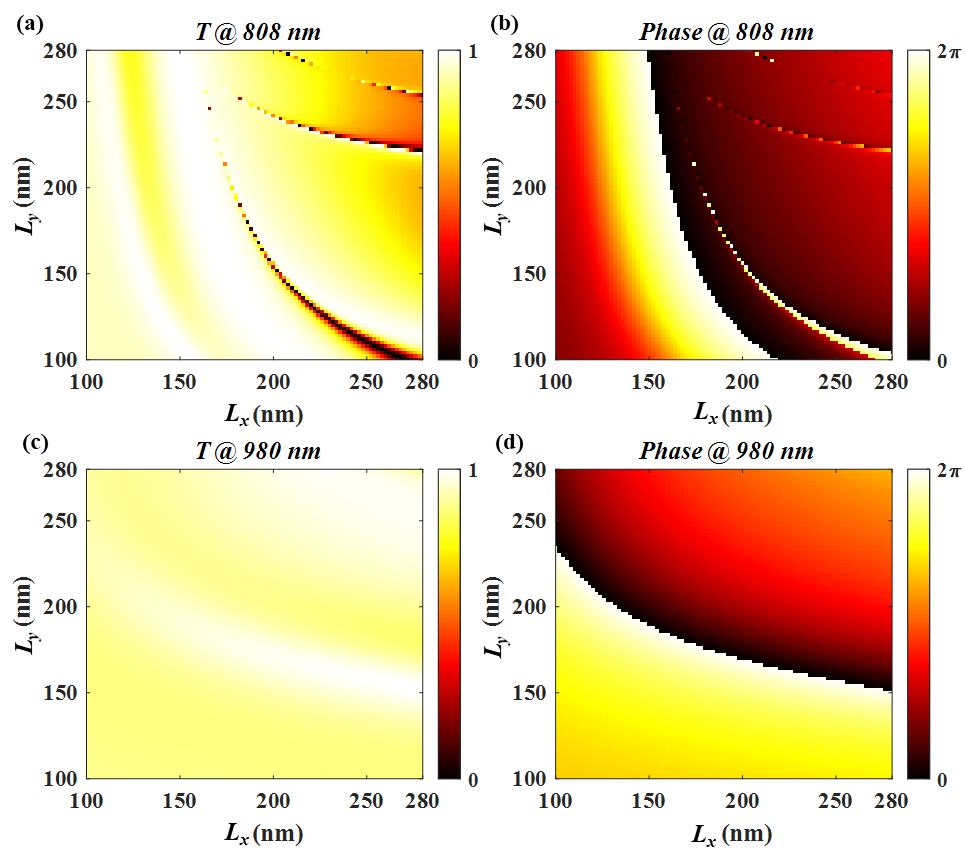


**Supplementary Figure 3.** Transmission response of metasurface units. The transmission is calculated when a unit of specified geometric parameters (illustrated in **Figure 2c**) is periodically arrayed. As width *L*_x_ and length *L_y_* of the silicon block inside the unit are ranged from 100 nm to 280 nm with fixed period length *P* = 350 nm and structural height *H* = 420 nm. (a) and (b) Transmissivity and phase as a function of the block size at wavelength 808 nm under x-polarized illumination, respectively. (c) and (d) Transmissivity and phase at wavelength 980 nm under x-polarized illumination, respectively. For y-polarized illumination, the optical response is similar to that in the case of x-polarized illumination due to the structural symmetry.

**Supplementary Note 4: Size of vortex spots**


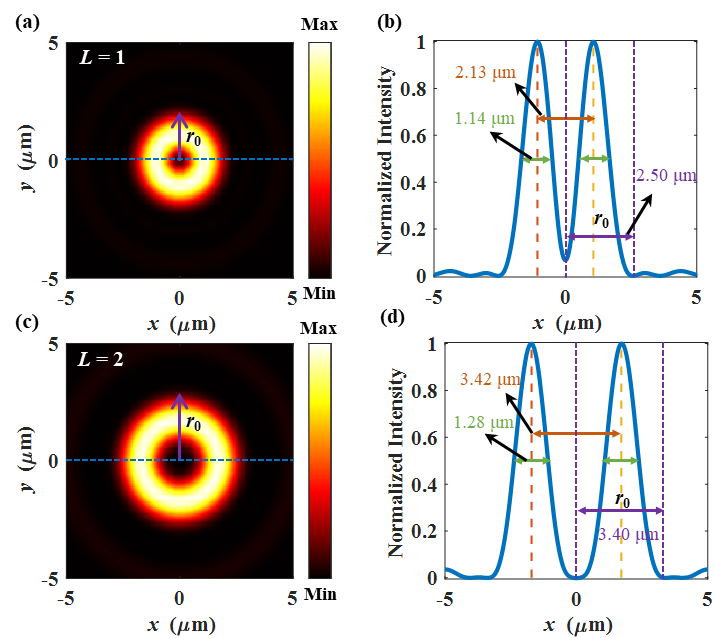


**Supplementary Figure 4**. Simulated vortex spots on the focal plane of a metasurface lens (radius 300 μm). The working wavelength is 980 nm. (a) and (c) Electric intensity profiles of vortex spots carrying topological charges *L* = 1 and *L* = 2, respectively. *r*_0_ denotes the minimum resolvable separation distance between adjacent vortices. (b) and (d) Cross-sectional electric intensity distributions along the dashed lines indicated in (a) and (c), respectively. As the topological charge increases, the size of the corresponding vortex spot expands.

**Supplementary Note 5: Boundary states of large vortex lattices**


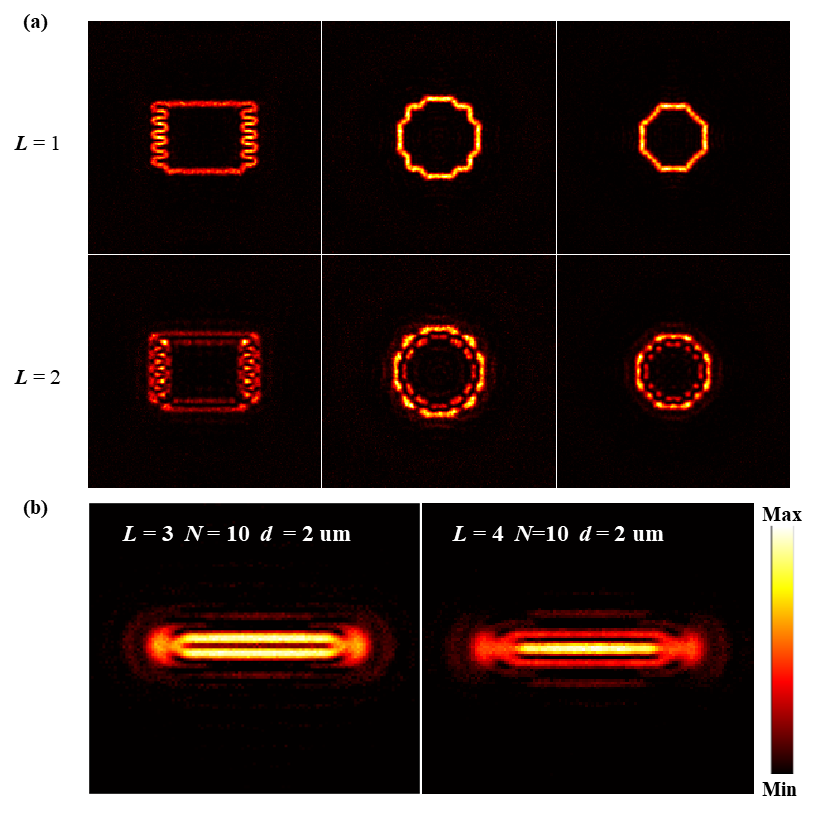


**Supplementary Figure 5**. Boundary states from STP. (a) Complex FVLs. Upper: topological charge *L* = 1. Lower: topological charge *L* = 2. (b) Long line chains of FVSs.

**Supplementary Note 6: Constructing parameters of FTCS**

**Table 1**

| Position    Wavelength | Focal length *f*_1_ = 300 μm | Focal length *f*_2_ = 400 μm |
| --- | --- | --- |
| 808 nm | $\left\{\begin{matrix} x_{0}(\xi)=10r(\xi)\cos(\xi) \\ y_{0}(\xi)=10r(\xi)\sin(\xi) \\ z_{0}(\xi)=f_{1} \\ r(\xi)=5-2\cos(5\xi) \\ \xi\in\left[0,2\pi\right] \end{matrix}\right.$ | $\left\{\begin{matrix} x_{0}(\xi)=15[\cos(\xi)-3\cos(3\xi)] \\ y_{0}(\xi)=15[\sin(\xi)+3\sin(3\xi)] \\ z_{0}(\xi)=f_{2}-15\sin(\xi) \\ \xi\in[0,2\pi] \end{matrix}\right.$ |
| 980 nm | $\left\{\begin{matrix} x_{0}(\xi)=10r(\xi)\cos(\xi) \\ y_{0}(\xi)=10r(\xi)\sin(\xi) \\ z_{0}(\xi)=f_{1} \\ \begin{matrix} r(\xi)=6-2\cos(6\xi) \\ \xi\in\left[0,2\pi\right] \end{matrix} \end{matrix}\right.$ | $\left\{\begin{matrix} x_{0}(\xi)=10[\cos(\xi)-2\cos(2\xi)] \\ y_{0}(\xi)=10[\sin(\xi)+2\sin(2\xi)] \\ z_{0}(\xi)=f_{2}-10\sin(\xi) \\ \xi\in[0,2\pi] \end{matrix}\right.$ |

**Supplementary table 1**. Constructing parameters of the FTCs shown in **Figure 5**.

**Supplementary Note 7: Designing a multi-channel multifocal metasurface**

As stated in the main text, the following phase-type transmission modulation is required in designing a multi-channel multifocal metasurface,

$$\begin{aligned} \varphi_{\lambda}^{x,y}\left( x,y \right)=arg\left[ t_{\lambda}^{x, y}\left( x,y \right) \right]\#\left( S1 \right) \end{aligned}$$

where the superscript and subscript are used to label different metasurface channels, including $\varphi_{808}^{x}\left( x,y \right)$, $\varphi_{808}^{y}\left( x,y \right)$, $\varphi_{980}^{x}\left( x,y \right)$, and $\varphi_{980}^{y}\left( x,y \right)$ (the unit of each number is nm). A practical metasuface cannot be completely independent in the four channels and the transmissivity is just close to one, thus one must make a compromise to realize an optimized imaging result. We adopt the following optimization way,

$$\begin{aligned} O_{808}^{x}\left( x,y \right)= {|\overline{A}}_{808}^{x}\left( x,y \right) e^{i\overline{\varphi}_{808}^{x}\left( x,y \right)} -e^{{i\varphi}_{808}^{x}\left( x,y \right)}| \#\left( S2 \right) \end{aligned}$$

$$\begin{aligned} O_{808}^{y}\left( x,y \right)= {|\overline{A}}_{808}^{y}\left( x,y \right) e^{i\overline{\varphi}_{808}^{y}\left( x,y \right)} -e^{{i\varphi}_{808}^{y}\left( x,y \right)}|\#\left( S3 \right) \end{aligned}$$

$$\begin{aligned} O_{980}^{x}\left( x,y \right)= {|\overline{A}}_{980}^{x}\left( x,y \right) e^{i\overline{\varphi}_{980}^{x}\left( x,y \right)} -e^{{i\varphi}_{980}^{x}\left( x,y \right)}|\#\left( S4 \right) \end{aligned}$$

$$\begin{aligned} O_{980}^{y}\left( x,y \right)= {|\overline{A}}_{980}^{y}\left( x,y \right) e^{i\overline{\varphi}_{980}^{y}\left( x,y \right)} -e^{{i\varphi}_{980}^{y}\left( x,y \right)}|\#\left( S5 \right) \end{aligned}$$

$$\begin{aligned} O\left( x,y \right)=\min\left( O_{808}^{x}\left( x,y \right)+O_{808}^{y}\left( x,y \right)+O_{980}^{x}\left( x,y \right)+O_{980}^{y}\left( x,y \right) \right)\#\left( S6 \right) \end{aligned}$$

Here, in each equation, the first item at the right side represents the practical transmission response of the designed metasurface with the amplitude near one, and the left side represents the error between the practical transmission and the required ideal transmission. The geometric parameters of metasurface unit (*x*, *y*) are finally determined to meet the minimum error required in Equation. S5 by scanning the built library in **Supplementary Figure 3**.

**Supplementary Note 8: Impact of the fabrication error and environment temperature on the imaging of a metasurface**

During the fabrication process, a 420 nm silicon layer is deposited onto a silica substrate based on the PECVD technology at first. The thickness and refractive index of the deposited layer are measured using an ellipsometer. Then, a required metasurface is fabricated. The primary source of the device error is attributed to the deviation in the lateral dimension of the nanostructure in each unit.

We investigate the influence of dimensional perturbation on the imaging quality across the four channels of the metasurface used in **Figure 5b** for demonstration. The maximum relative dimensional variation along the width direction is denoted as *δ*_x_ = ∆*L*_x_/*L*_x_, and that along the length direction as *δ*_y_ = ∆*L*_y_/*L*_y_, where ∆*L*_x_ and ∆*L*_y_ represent the width and length perturbations, respectively. Random perturbation is applied to each unit of the metasurface. For two formed *m*×*n* images of I (reference image) and K (distorted image), the mean squared error (MSE) is defined as

$MSE= \frac{1}{mn}\sum_{i=1}^{m} \sum_{j=1}^{n} \left[ I\left( i,j \right)-K\left( i,j \right) \right]^{2}$ (S7)

where *I*(*i*, *j*) and *K*(*i*, *j*) represent pixel intensity values. Then, the peak signal-to-noise ratio (PSNR) of the distorted image is defined as

$PSNR=20\log_{10} \frac{I_{\max}}{\sqrt{MSE}}$ (S8)

where *I*_max_ represents the maximum possible pixel value. Since we have normalized the images in our calculation, *I*_max_ = 1. A higher PSNR value as a performance metric indicates a smaller difference between the distorted and reference images (less distortion and better image quality). The statistical PSNR distribution obtained by varying the maximum relative perturbation magnitude is summarized in **Supplementary Figure 6**, used to evaluate the imaging robustness of the metasurface against the fabrication error. When the maximum relative dimensional error remains within 15%, the PSNR values for all the four channels exceed 30 dB, indicating robust imaging performance. Notably, the PSNR values in channels 1 and 3 surpass 40 dB, displaying superior image quality as compared to channels 2 and 4. The 3D noncoplanar focal knotted curves exhibit more obvious sensitivity to the dimensional perturbation.


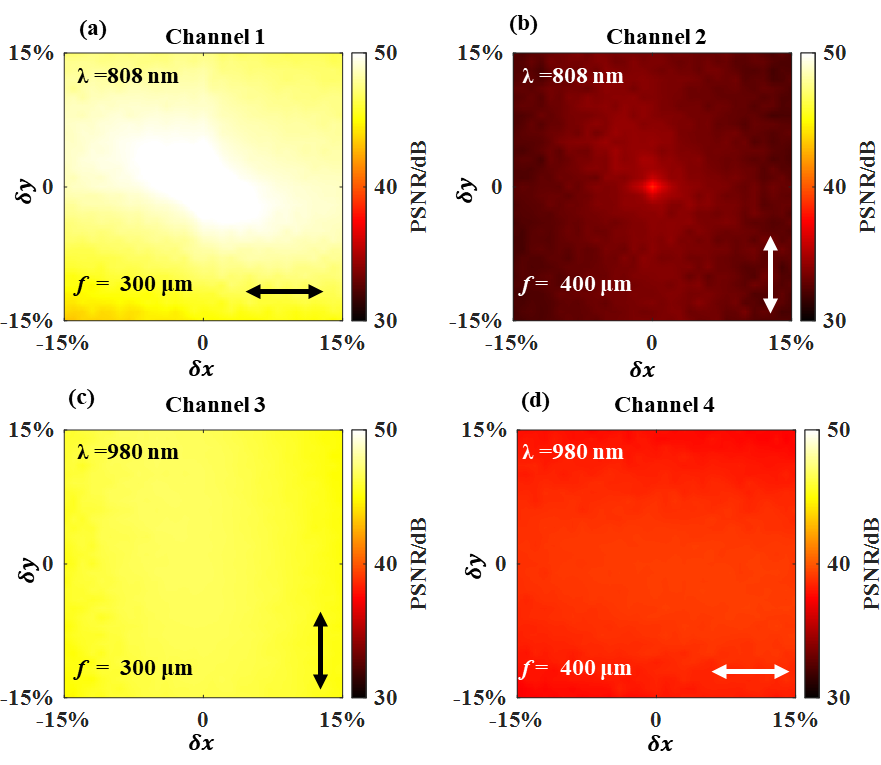


**Supplementary Figure 6**. Imaging robustness of a metasurface against the fabrication error. The metasurface used in Figure 5b is investigated for demonstration, and the four channels 1-4 are labelled in the figures.

Different external environmental factors also impact the imaging quality of a metasurface. For example, silicon possesses a positive thermo-optic coefficient of d*n*/d*T* = 1.85×10^-4^ K^-1^ (*n* and *T* are the silicon index and the temperature, respectively)^[1, 2]^. We numerically calculate the impact of the temperature on the imaging quality of the metasurface used in **Figure 5b** based on the PSNR metric. The PSNR values for the four channels of the metasurface are shown in **Supplementary Figure 7** when the temperature is increased from 25 ℃ to 40 ℃ gradually. The imaging quality across all the four channels is gradually degraded, but not seriously deteriorated. The two channels at wavelength 808 nm (channels 1 and 2) are more sensitive than those at wavelength 980 nm (channels 3 and 4). This is because the variation of the silicon index imposes stronger impact on the response of the silicon brick in each unit of the metasurface at a shorter wavelength.

**Supplementary Figure 7.** Impact of the environmental temperature on the imaging quality of the metasurface used in Figure 5b.

**Supplementary Note 9: Fluorescence emission of UCNPs**

**Supplementary Figure 8**. Fluorescence emission of UCNPs under 980 nm laser excitation.

**Reference：**

[1] SHIN M J, BAN Y, YU B-M, et al. Parametric characterization of self-heating in depletion-type si micro-ring modulators [J]. IEEE Journal of Selected Topics in Quantum Electronics, 2016, 22(6): 116-22.

[2] KOMMA J, SCHWARZ C, HOFMANN G, et al. Thermo-optic coefficient of silicon at 1550 nm and cryogenic temperatures [J]. Applied Physics Letters, 2012, 101(4): 041905.
